# Evaluating the Acceptability of Using Virtual Reality to Promote Physical Activity Among Latino, Latina, and Latine Adults With Cardiometabolic Risk Factors and Obesity in Underresourced Settings: Protocol for a Qualitative Focus Group Study

**DOI:** 10.2196/80534

**Published:** 2026-01-12

**Authors:** Desiree R Acosta, Leslie Aguilar-Hernandez, Gael Perez, Iris Y Guzman-Ruiz, Josyel M Castellon, Jailene Cruz, Liana Gutierrez, Paulina Monteon-Garcia, Vanessa N Torres, O Kenrik Duru, Yelba Castellon-Lopez

**Affiliations:** 1 Program in Medical Education-Leadership and Advocacy (PRIME-LA), David Geffen School of Medicine at UCLA, Los Angeles, CA, United States; 2 Cancer Research Center for Health Equity, Cedars-Sinai Medical Center, 8700 Beverly Blvd, Los Angeles, CA, 90048, United States, 1 4243150026; 3 General Internal Medicine-Health Services Research, David Geffen School of Medicine at UCLA, Los Angeles, CA, United States

**Keywords:** virtual reality, physical activity, dance, acceptability, health equity, Latino, Latina, Latine

## Abstract

**Background:**

Obesity represents a significant public health challenge in the United States, particularly among Latino, Latina, and Latine communities and those in underresourced settings. Virtual reality (VR) is a new and innovative technology that can promote physical activity and has the potential to overcome some structural barriers. However, there are few studies that explore the acceptability of using this new technology among high-risk groups in underresourced settings.

**Objective:**

We outline a community-informed protocol for conducting focus groups with Latino, Latina, and Latine adults who have cardiometabolic risk factors and obesity residing in underresourced communities. The focus groups will assess the acceptability of a culturally aligned VR program to promote physical activity.

**Methods:**

Using a community-engaged approach informed by community health workers and a community advisory board, we delivered an immersive VR dance experience to Latino, Latina, and Latine adult participants with cardiometabolic risk factors and obesity. Following the VR experience, we conducted semistructured focus group interviews to assess acceptability guided by the theoretical framework of acceptability. Data collection included a baseline demographic survey and focus group discussions to evaluate participant experiences and the VR program’s acceptability.

**Results:**

As of April 2025, we have completed 7 focus groups with 44 participants across 3 age groups: 18 to 29 years, 30 to 49 years, and 50 to 70 years. Data collection was completed in May 2025, and study findings are expected to be published in February 2026.

**Conclusions:**

The fully analyzed data from this study will offer insights into leveraging VR as an innovative tool for promoting physical activity in underserved populations, contributing to the broader literature on digital health equity and obesity prevention.

## Introduction

Obesity is a major health concern in the United States as it significantly increases the risk of various chronic diseases, including heart disease, stroke, type 2 diabetes, and cancer [1]. Data from the National Health and Nutrition Examination Survey show that 40.3% of adults in the United States were classified as obese from August 2021 to August 2023 [2]. Obesity prevalence varies significantly based on race and ethnicity, with higher rates among Latino, Latina, and Latine adults than among White adults [3]. Estimates from 2017 to 2018 show that 44.8% of Latino, Latina, and Latine adults and 50.4% of Mexican American adults were classified as obese compared to 42.4% of non-Hispanic White adults [4]. Furthermore, the prevalence of obesity is higher among American adults with lower educational attainment and lower household income—with the lowest prevalence observed among those with a bachelor’s degree or higher [2,5,6]. Latino, Latina, and Latine adults experience a higher prevalence of obesity, which is accompanied by a disproportionate burden of obesity-related chronic diseases such as type 2 diabetes and certain cancers linked to obesity [4]. As Latino, Latina, and Latine adults represent one of the fastest-growing ethnic minority groups, and currently the largest ethnic minority group, in the United States, there is a pressing need for population-specific public health strategies to mitigate the health impacts of obesity-related diseases [7].

Physical inactivity and sedentary lifestyles are key contributors to obesity [8]. Between 2017 and 2020, only 25.3% of adults in the United States reported being physically inactive outside of work [9]. Among Latino, Latina, and Latine adults, this figure was even higher, with 31.7% reporting no physical activity outside of work, resulting in an increased risk of obesity-related chronic diseases [9]. Racial and ethnic disparities in physical activity may be influenced by structural barriers, including lack of access to safe spaces for exercise, walkable neighborhoods, and sufficient time [9,10]. These findings underscore the urgent need for targeted, culturally responsive interventions that can overcome barriers and address obesity, physical inactivity, and sedentary lifestyles within the Latino, Latina, and Latine community [11]. This population encounters a unique combination of cultural, environmental, and structural challenges that standard obesity prevention and physical activity interventions frequently overlook. Language and cultural norms related to diet, family roles, and food traditions can influence preferences and behaviors. Unfortunately, many interventions are not tailored to address these important elements [4,11]. A systematic review of culturally adapted interventions revealed that incorporating bilingual or bicultural staff, involving family, and implementing community-based formats showed better engagement and outcomes among Latino, Latina, and Latine participants compared to nonadapted interventions [12,13]. These disparities and contextual barriers highlight the need for culturally responsive interventions tailored to Latino, Latina, and Latine adults to enhance acceptability, engagement, and effectiveness.

Therapeutic virtual reality (VR) is gaining popularity as a health-promoting strategy to enhance physical activity and exercise [14]. Through a head-mounted display and a close-proximity screen, VR can transport users into a lifelike, 3D experience [15,16]. A VR exergame is an interactive exercise-based game that uses VR to immerse users in a movement-driven experience. By integrating motion tracking and gamification, VR exergames encourage physical activity through engaging scenarios such as sports, dancing, or fitness challenges. These games have been shown to enhance intrinsic motivation, self-efficacy, and physical activity enjoyment and adherence in adolescents and adults [17-21]. Dance- and music-based exergames have been highly rated [22] and show promise in promoting physical activity [23]. Furthermore, qualitative studies have demonstrated that older adults often prefer exergames and dance-based VR experiences over traditional, less interactive physical activity programs, citing greater motivation and perceived benefits (eg, cognitive and psychological), in both general and underserved settings [22,24,25]. VR-based exergaming has been shown to target key drivers of long-term physical activity engagement. For example, a review of 29 studies concluded that exergaming not only enhances enjoyment but can also elicit moderate to vigorous exercise intensity and reduce perceived exertion, compared to traditional exercise formats, indicating that exergames encourage participants to engage in exercise of moderate to vigorous intensity compared to other exercise programs [26,27]. Beyond physical exertion, VR exergaming has also demonstrated potential for cognitive and emotional benefits among older adults, including moderate improvements in cognitive function, memory, and depressive symptoms, suggesting advantages for both mental health and healthy aging [27-29]. However, despite the promise of VR-based exergaming, there are few studies exploring the acceptability of VR among Latino, Latina, and Latine groups with cardiometabolic risk factors and obesity. Addressing this gap is essential to ensure equitable benefits from VR-based interventions that have demonstrated potential to promote physical activity and improve health outcomes.

By enabling individuals to engage in physical activity from the safety and convenience of their homes, VR technology has the potential to reduce barriers and enhance physical activity access in the Latino, Latina, and Latine community. This paper describes a community-informed protocol (version 1) for the Movement Over Virtual Reality study, which aims to assess the acceptability of a VR dance-based exercise program to promote physical activity among Latino, Latina, and Latine adults with cardiometabolic risk factors and obesity.

This protocol was developed in consultation with community health workers (CHWs) from the Latino, Latina, and Latine population and a community advisory board (CAB). If the focus groups demonstrate the acceptability of using VR to promote physical activity among Latino, Latina, and Latine adults with cardiometabolic risk factors and obesity, particularly those in underresourced settings, we plan to conduct a future pilot study to assess the feasibility of at-home VR exercise by providing participants with VR headsets and wearable devices to track use over time.

## Methods

### Pilot Phase With CHWs

To refine the Movement Over Virtual Reality focus group study protocol, we conducted 2 pilot sessions with 24 CHWs to gather feedback on our proposed study procedures. All CHWs were Spanish-speaking Latino, Latina, and Latine adults between the ages of 30 and 70 years, as well as first-time VR users. The CHWs work independently to engage communities and provide health education, advocacy services, and support. During the 2 pilot sessions, CHWs were paired with trained Spanish-speaking facilitators at a ratio of 1 instructor to 3 CHWs to provide instruction on headset placement, VR hand controller use, and the proposed VR experience. The initial VR experience involved CHWs completing skill-building tasks such as hitting targets to become familiar with VR hand-body coordination and was followed by a dance session with a virtual dance partner. Facilitators remained with participants throughout the VR experience to ensure safety and answer technical questions. After the 30-minute experience, we debriefed CHWs on how to improve future focus group sessions. CHWs emphasized the need for 1:1 facilitation and suggested adding a group VR orientation session, separate “stations” with physical boundaries, and a nearby chair for those who may experience dizziness or fatigue, as well as shortening the session by removing skill building and proceeding directly to the dance experience. Participants also requested a Spanish version of the VR dance experience. All suggestions were integrated into this study protocol except for a fully Spanish-language VR dance experience due to lack of availability on the Meta platform. CHWs were compensated with US $100 for their support in providing feedback to refine the study protocol.

### Incorporating CAB Feedback

A CAB composed of community leaders and the research study team convenes quarterly, and CAB members receive an honorarium of US $125 per hour funded as part of a larger career development grant (K23DK129828). We held 2 meetings to review and adapt study recruitment materials and focus group questions. During these meetings, the study team presented the theoretical framework of acceptability and the proposed focus group interview guide, which included questions in both English and Spanish [24]. The CAB provided recommendations to modify focus group questions to incorporate more lay language and enhance Spanish interpretation. Additionally, the study team reviewed flyers and the step-by-step recruitment approach, resulting in changes to the recruitment materials. These changes included adding a hyperlink and web address to the screening survey alongside the QR code and a phone number for interested participants to call.

### Eligibility Criteria for Focus Group Participants

Eligibility criteria for focus group participants were (1) self-identification as Latino, Latina, and Latine; (2) age of ≥18 years; and (3) ability to speak English or Spanish. Given that obesity is a major risk factor for type 2 diabetes [1,5], cardiometabolic risk factors were screened based on a self-reported diagnosis of prediabetes or a moderate to high risk score for developing type 2 diabetes based on the Centers for Disease Control and Prevention’s “Do I Have Prediabetes?” screening tool [1,30]. Individuals with physical injuries preventing them from engaging in 30 minutes of physical activity were excluded. Prior VR experience was not required for participation.

### CHW-Led Recruitment

**Figure 1.**
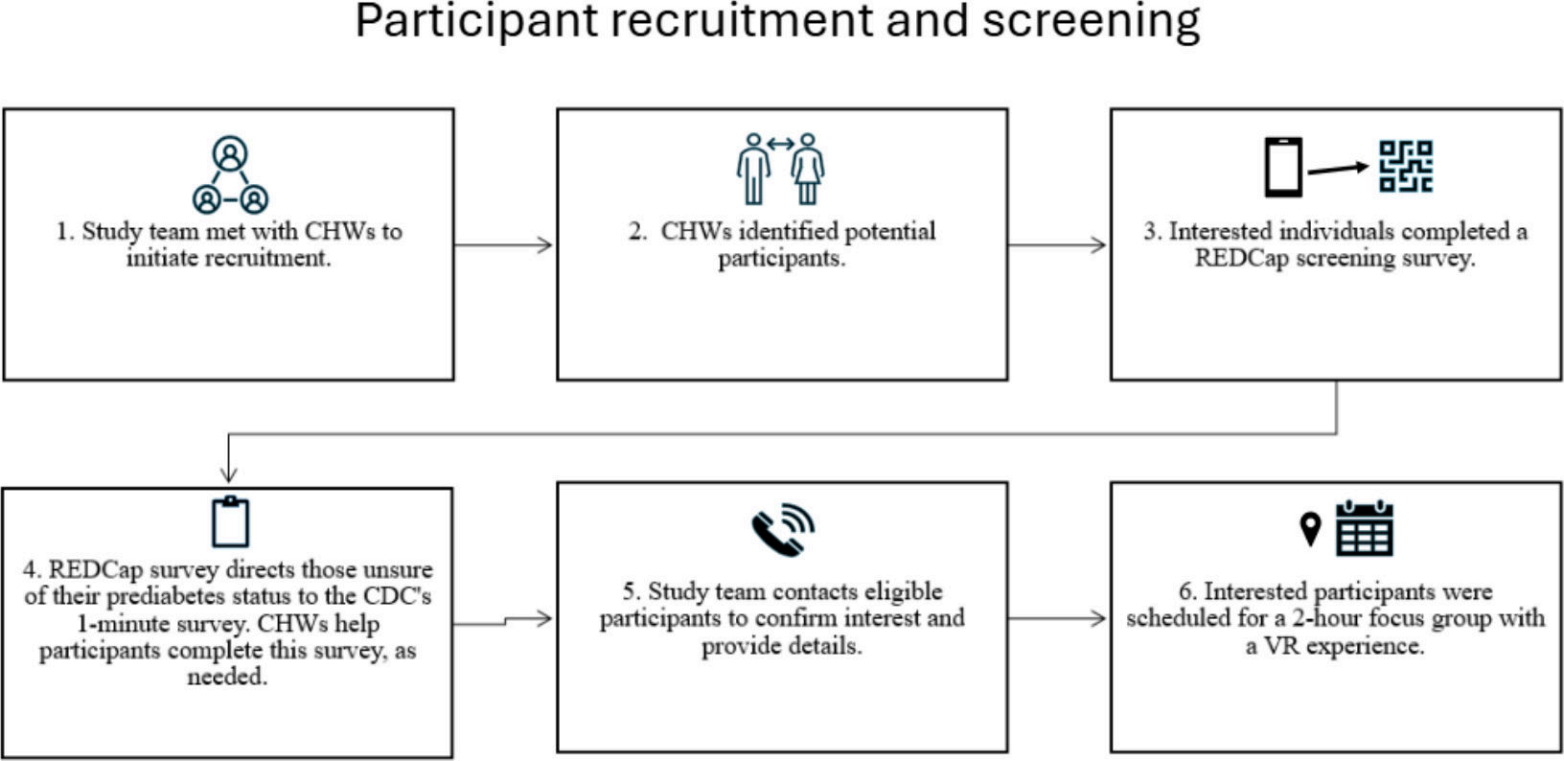
Flowchart of the study participant recruitment and screening process. CDC: Centers for Disease Control and Prevention; CHW: community health worker; REDCap: Research Electronic Data Capture; VR: virtual reality.

Participants were recruited in collaboration with CHWs (Figure 1) in south and east Los Angeles, California, as these are 2 underresourced areas with predominantly Latino, Latina, and Latine populations [31,32]. Recruitment efforts were led by the study team and trained CHWs culturally and linguistically aligned with the anticipated study population. The CHWs spoke English and Spanish and were aged between 30 and 70 years and of a similar cultural background to that of the target study participants. Most CHWs reported prior experience working on research studies and providing health education, resource linkages, and service navigation. Before commencing recruitment, the CHWs received in-person training, in which they were briefed on the study and their expected role in recruitment. After completing the training, 8 CHWs expressed interest in participating, with the goal of recruiting 6 to 8 participants each over a 5-month period. A memorandum of understanding outlining the study goals, the study team commitments, and expectations for CHWs was provided to those amenable to recruiting for the study. CHWs were supplied with recruitment materials, including study flyers, a 1-page frequently asked question sheet, and a Meet the Study Team document based on their recommendations. CHWs were compensated with US $250 for their support with recruitment.

Throughout recruitment, the study team held 30-minute biweekly virtual meetings with the CHWs. During these meetings, the study team provided recruitment and enrollment updates. Additionally, the study team created a WhatsApp group chat to provide reminders about virtual meetings, aggregate recruitment updates, and address general questions for the group. No personal identifiers or individual-level information were shared on the WhatsApp platform. The study team was also available to answer CHWs’ individual questions or concerns via phone and email. Furthermore, the study team also fostered continual engagement by providing monthly workshops on health topics selected by the CHWs, including preventive women’s cancer screenings and educational sessions on type 1 and 2 diabetes.

### Study Procedures

Upon identifying potential participants, CHWs offered them a QR code or link to a brief eligibility survey, which was received by the study team once it was completed by potential participants. Screening surveys were collected and managed using the secure web-based software platform REDCap (Research Electronic Data Capture; Vanderbilt University) [33,34]. Bot spam was prevented by requiring participants to complete a reCAPTCHA (reCAPTCHA Inc) test before accessing the survey. Participants provided their contact information and age group and confirmation that they had been diagnosed with prediabetes within the previous 12 months. Participants who did not have a prediabetes diagnosis or were unsure of it were asked to complete the Centers for Disease Control and Prevention’s “Do I Have Prediabetes?” screening tool through an embedded link to determine their prediabetes risk score. Participants scoring 0 to 4 were considered low risk and ineligible, whereas scores of 5 to 10 were considered moderate to high risk and eligible for study inclusion. The interest survey and risk tool were available in both English and Spanish. Eligible individuals were contacted by the study team via phone or email to schedule their participation in an in-person focus group in their preferred language (ie, English or Spanish).

### VR Dance Experience and Focus Group Procedures

#### VR Equipment

To facilitate the VR dance experience, we used the Meta Quest 3S VR headset (Reality Labs), which includes head and hand position tracking for accurate spatial orientation. Participants interacted with the VR environment using 2 wireless Meta Quest Touch Plus controllers (Reality Labs), which included wrist straps to prevent slippage. These controllers served as motion sensors, enabling precise real-time tracking of participants’ full-body movements. We used FitXR as the VR fitness application because it offered an interactive Zumba dance experience with Spanish subtitles. Specifically, participants engaged in a 10-minute beginner Zumba session set to Latin music led by a virtual instructor. To support the VR setup, a Netgear hot spot offering 5G network compatibility and encrypted security to support multiple devices was used to provide wireless internet connectivity.

#### Stations for the VR Dance Experience

VR sessions took place in a large room in a community center. Each participant was assigned to a “station,” which served as the designated area where they could safely engage in the immersive VR dance experience. To promote safety, stations were spaced at approximately 2 arms’ length. The study staff used visual boundaries on the floor to create virtual safety boundaries for each VR headset—a built-in safety feature notifying VR users when they approached the boundaries of their area.

Each station was labeled with a number corresponding to the participants’ assigned headset. A printed checklist was also displayed at each station for facilitators to reference during setup and throughout the session. Additionally, each station featured a centrally located chair to accommodate participants preferring to be seated during the VR dance experience or who experienced motion sickness or disorientation during their session.

#### Participant Orientation for the VR Dance Experience

Before beginning the VR dance experience, the study team provided a structured orientation to prepare participants that incorporated CHW feedback from the pilot sessions. Participants received orientation materials in Spanish or English based on their preference, including a slide presentation with a detailed agenda, introduction to study staff, consent forms, pre- and postsurvey instructions, and an overview of the VR equipment. The slide presentation included videos and images to review basic VR skills, including how to adjust the VR headsets; use the hand controller buttons; and set physical space boundaries, a feature integrated into the VR technology to maintain user safety.

#### 1:1 VR Facilitation

Participants were paired with a culturally and linguistically congruent study team member who guided and supported them throughout the VR dance experience. All VR facilitators received training on VR equipment operation and navigation of the VR dance program to ensure effective assistance of participants. At the start of the VR dance experience, each facilitator introduced themselves to their assigned participant and accompanied them to their designated VR station. The facilitator then assisted participants in putting on the VR headset. Each facilitator checked that participants’ VR headsets were secure and comfortable and the proper placement of controller wrist straps to prevent accidental drops during the experience. Once participants indicated readiness, the facilitators launched the VR dance program, remaining with the participants throughout to ensure continued safety and provide real-time support. To better assist participants, facilitators also paired VR headsets with a secondary device (eg, phone or tablet) to mirror participants’ VR display. This allowed facilitators to respond to questions while seeing participants’ mirrored real-time experience in the headset. At the end of the experience, facilitators aided in removing the VR equipment and reviewed VR performance metrics with the participants, including their overall score, best streaks, and points earned.

#### Data Collection

Participants completed a baseline questionnaire after consenting to study participation and before the VR experience to assess their previous VR use. After the VR experience, participants completed a demographic survey including their age, gender, educational attainment, income, marital status, employment status, country of birth, and preferred language. The survey also included questions about participants’ internet and technology use; neighborhood composition; and self-rated health, sleep quality, and perceived stress levels. Administering the demographic survey after the VR session improved workflow efficiency by allowing participants who finished earlier to begin the survey while others completed their VR dance experience.

Next, focus groups were conducted in person by a primary facilitator and a cofacilitator (both trained study team members) using an interview guide developed in collaboration with the CAB and CHWs. The semistructured interview guide included a standard introduction; ground rules; and a series of open-ended questions designed to elicit participants’ perspectives on the VR dance experience and the acceptability of VR as a physical activity modality among Latino, Latina, and Latine adults (see Multimedia Appendix 1). A third study team member took notes to guide future transcript analysis. Focus group discussions were audio recorded using digital recorders. All recordings were transcribed verbatim, and when necessary, a certified Spanish translation and transcription service was used to translate them into English to ensure accuracy. Each focus group lasted approximately 1 hour, allowing for sufficient discussion time. Finally, we administered postintervention surveys immediately after the focus groups. The survey asked participants to rate their satisfaction with their VR dance and overall VR experience. To assess safety, participants were also asked whether they experienced any side effects during VR use. All data were collected in the participants’ preferred language and deidentified for privacy.

### Data Analysis

Focus group interviews will be coded using Dedoose (SocioCultural Research Consultants), a qualitative data software platform. The study team will develop a coding framework based on the semistructured interview guide informed by the theoretical framework of acceptability, which evaluates the perceptions and attitudes of participants regarding the intervention [35]. Focus group transcripts will be independently coded by 2 trained research team members using a predefined coding framework in alignment with the coding reliability thematic analysis approach. Each week, the study team will meet to review and refine the preliminary codebook. Once finalized, subsequent transcripts will be coded in pairs. Coder discrepancies will be discussed among the research team and resolved through consensus. Field notes will be used to monitor focus group data for saturation. The analysis of the transcripts and coding will continue until no new themes emerge from the data. To arrive at the final themes and subthemes, we will achieve consensus through iterative discussions among research team members, and this process will be documented to ensure thoroughness. Data will be analyzed using thematic analysis, a systematic method for identifying, analyzing, and reporting patterns within the data [36]. Triangulation will be achieved by comparing focus group data with field notes taken during the sessions and other data sources such as the demographic survey and postintervention survey open-ended questions.

### Ethical Considerations

This study was approved by the Cedars-Sinai Institutional Review Board (IRB; protocol ID: STUDY00003356). All procedures involving human participants were conducted in accordance with the Cedars-Sinai IRB. Written informed consent was obtained from all participants before initiating the study by trained study staff. Participant data collected during the focus groups were deidentified and stored securely to maintain confidentiality. A US $100 gift card was given to each participant who completed the study as compensation for their time and contributions. No copyrighted surveys were used in this study. This study did not involve research on animals.

## Results

This study was funded on July 1, 2024, and received IRB approval on August 9, 2024. Participant recruitment began in January 2025. As of April 2025, a total of 125 individuals have been screened, and 35.2% (44/125) of participants have been enrolled. Seven focus groups have been conducted with enrolled participants across 3 age groups: 18 to 29 years, 30 to 49 years, and 50 to 70 years. Five focus groups were conducted in Spanish, and 2 were conducted in English. Data collection was completed in May 2025, and preliminary analyses began shortly thereafter.

A total of 73% (32/44) of the participants enrolled as of April 30, 2025, identified as female, with an average age of 46 (SD 14.1, range 18-72) years. Most participants (27/44, 61%) preferred Spanish, and 82% (36/44) reported being born outside of the United States (Table 1). Study findings are expected to be published in February 2026 and will be disseminated to the broader scientific community as well as our partner CHWs, the CAB, and the participants.

**Table 1.** Characteristics of the focus group participants (n=44).

Participant demographics	Values
Age (y), mean (SD)	46 (14)
Categorical age (y), n (%)	
18‐29	9 (21)
30‐49	16 (36)
50‐70	19 (43)
Sex, n (%)	
Male	12 (27)
Female	32 (73)
Preferred language, n (%)	
Spanish	27 (61)
English	10 (23)
Both	7 (16)
English proficiency, n (%)	
Very well	10 (23)
Well	4 (9)
Not well	23 (52)
Not at all	3 (7)
Missing/Prefer not to answer	4 (9)
Ethnicity^a^, n (%)	
Mexican, Mexican American, or Chicano	25 (57)
Salvadoran	3 (7)
Guatemalan	11 (25)
Nicaraguan	2 (5)
Indigenous	1 (2)
Other Hispanic, Latino, or Spanish origin	6 (14)
Born outside of the United States, n (%)	36 (82)
Country of birth, n (%)	
Mexico	18 (50)
Guatemala	9 (25)
El Salvador	3 (8)
Other^b^	4 (11)
Prefer not to respond	2 (6)
Time lived in the United States (y), mean (SD)	27 (16)
Household size, n (%)	
0‐4	27 (61)
5‐6	15 (34)
Prefer not to respond	2 (5)
Employment status, n (%)	
Employed	17 (39)
Unemployed	4 (9)
Other^c^	23 (52)
Household income (US $)^d^, n (%)	
<41,600	20 (46)
41,600‐69,350	8 (18)
>69,350	3 (7)
Prefer not to respond	13 (29)
Marital status, n (%)	
Married	18 (41)
Other	26 (59)
General health status, n (%)	
Excellent	0 (0)
Very good	8 (18)
Good	12 (27)
Fair	22 (50)
Poor	2 (5)
Diagnosis of prediabetes, n (%)	
Yes	33 (75)
No	7 (16)
Not sure	4 (9)

a Participants were allowed to select more than 1 option.

b Includes Colombia, Jamaica, Nicaragua, and Peru.

c Includes “housekeeper,” “retired,” “disabled,” “temporarily laid off,” “student,” “caregiver,” and “other.”

d Levels based on the US Department of Housing and Urban Development in Los Angeles County based on a family size of 4 persons in 2024.

## Discussion

This paper describes a protocol for evaluating the acceptability of a Latin dance–inspired VR exergame aimed at promoting physical activity among Latino, Latina, and Latine adults with cardiometabolic risk factors and obesity in underresourced settings. Our objective is to identify potential barriers and share insights relevant to implementing a technology-driven study in these environments, with a focus on ensuring access for groups that may otherwise lack exposure to new technology. Furthermore, our community-informed protocol may support other research that addresses issues related to digital health equity.

To address these challenges, this study protocol incorporated practical solutions developed with input from CHWs who reflect the study population and were involved in the early stages of protocol development. On the basis of our experience, individualized guidance from trained staff members who were culturally and linguistically aligned with participants was a key component for first-time VR users. In addition, as many participants were first-time VR users with limited digital literacy, we provided clear, hands-on instructions, including how to adjust the headset, use the controllers, and navigate safety features such as the virtual boundary system.

CHWs were crucial in refining the protocol and recruiting participants, leveraging their trust and community ties to engage populations traditionally underrepresented in research [37-39]. Furthermore, CHW involvement offers a sustainable strategy for addressing digital health access and literacy disparities that should be considered [40-42]. To sustain CHW engagement over the 5-month recruitment period, we held biweekly meetings to share recruitment updates and outreach strategy collaboration. Additionally, we fostered a bidirectional colearning environment through health education workshops on topics selected by the CHWs. Working with CHWs to use the VR equipment and participate in the pilot VR sessions before participant recruitment was key to building trust and providing them with the opportunity to share information about the study authentically as part of their recruitment approach.

On the basis of our experience, there are some potential challenges to consider when conducting technology-driven studies with populations with limited or no prior VR experience. These include access to facilitators who can help participants with headset operation and troubleshooting of any technical issues that may arise. Space considerations should include ample room to account for participant movement during headset use, as well as strong Wi-Fi signal to support the simultaneous use of multiple devices. Our decision to implement a short session was influenced by the need to allocate additional time to preparing the VR headsets at study onset. This included setting up generic profiles on the headsets, allowing participants to experience the program without the need to create individual profiles and avatars. While this approach limits personalization, preventing participants from tailoring their experience to their preferences and fitness levels, it was necessary to streamline the process and enable participants to focus on the VR experience itself. Economizing time and allowing participants to focus on their experience of engaging with the headset outweighed this limitation.

If the proposed qualitative findings demonstrate high acceptability within the target population, the next phase will involve conducting a longitudinal feasibility study. In this future study, participants will be provided with a VR headset to use at home, allowing us to measure physical activity and behavioral outcomes and assess long-term adherence over time. Although this study focuses on evaluating the initial acceptability of the VR dance-based exercise program, future research should incorporate a control group. This addition will facilitate a more rigorous assessment of the efficacy of VR interventions on physical activity levels over time. In addition, we propose the use of wearable technology (ie, Fitbit trackers) in future studies as an objective measure of physical activity. Future studies should aim to equitably address technological literacy and meet the infrastructural needs of resource-limited settings by considering solutions such as providing portable Wi-Fi hot spots, as used in this study. Additionally, identifying or developing a Spanish-language option for a VR dance program should be prioritized to better meet the needs of participants. In this study, we selected the FitXR program because it features Spanish subtitles. It was also the most straightforward to follow with minimal instruction among the available options. However, the limitation of not having a full Spanish-language VR program available may create inequities for those whose preferred language is Spanish. This is an important area to address in future research. Expanding recruitment beyond south and east Los Angeles communities will also be crucial to enhance the generalizability of the findings across diverse Latino, Latina, and Latine populations, ensuring broader applicability and impact.

Findings from this and future studies will provide insight and may serve as a step toward ensuring equitable access to innovative technology-driven health interventions, including VR, for all communities. Future longitudinal studies should explore innovative models that support the sustainable use of VR in both clinical and community settings. By leveraging gamification technology, these models have the potential to promote healthy behaviors and physical activity in high-risk groups through technology-driven approaches. Similar models have been successful, as demonstrated by Medicare’s coverage of VR as evidence-based treatment for pain management and mental health, illustrating the potential to integrate cutting-edge technology as a health care benefit [43,44].

## Supplementary material

10.2196/80534 Multimedia Appendix 1 Institutional review board–approved focus group question guide.

10.2196/80534 Checklist 1 SPIRIT checklist.

## Abbreviations

CAB: community advisory board
CHW: community health worker
IRB: institutional review board
REDCap: Research Electronic Data Capture
VR: virtual reality
