# Supplementary material for: Evaluating the Acceptability of Using Virtual Reality to Promote Physical Activity Among Latino, Latina, and Latine Adults With Cardiometabolic Risk Factors and Obesity in Underresourced Settings: Protocol for a Qualitative Focus Group Study
Source: JMIR Res Protoc. 2026 Jan 12;15:e80534. doi: 10.2196/80534 (PMC12795479; doi:10.2196/80534)
Supplement: Multimedia Appendix 1 [file resprot-v15-e80534-s001.pdf]

<u>Welcome</u>

Facilitator: "Good afternoon, everyone. My name is ___________ and I will be facilitating our discussion today. I want to thank each one of you for joining us. This focus group will help us understand if a virtual reality (VR) experience with a Latin dance theme would encourage people in the Latino community who have prediabetes or are at high risk of prediabetes to move more. Your feedback is very valuable and will help determine how this program is developed and implemented.

<u>Introduction to Topic</u>

Facilitator: "Today we are here to talk about your thoughts and feelings on using Virtual Reality technology as a tool to increase physical activity in the Latino community. We are particularly interested in your perspectives on its use in programs designed to prevent diabetes. Before we start, we want to set a few rules before we start the conversation.

1. There are no right or wrong answers.
2. Please dont respond with head movements, such as nodding or shaking your head. Please voice your opinion so that the recorders can capture it.
3. Please don't use any identifiers when speaking, such as your name to maintain confidentiality.
4. We may have different opinions and that is okay. Please feel free to express your true opinions.

<u>Discussion Questions</u>

How the person feels about the intervention

1. How do you feel about the virtual reality (VR) experience you had today?

2. What did you like about it? What did you not like?
   a. Why?

Barriers to participate in the intervention

1. How easy or how difficult was it for you to participate in VR?

2. How was your experience with the VR equipment?
   a. Was there any difficulty with the VR equipment?

3. Now that you have participated in VR, what kind of supports would be needed to participate in a VR program like this at home?

Ethicality (The Extent To Which An Intervention Has Good Fit With An Individuals Or Communities Value System)

1. Do you think that VR programs for people who struggle to do exercise could be used in the Latino/a community? Is it aligned with the values of the community?

2. What are your thoughts about using a VR program with Latin Dance to motivate people to do physical activity? How is that aligned with the community?
3. If you could change one thing about the VR program to make it more aligned with Latino culture, what would it be?
4. Do you think that a VR program in which people dance to do exercise is relevant to men? Why or why not?

Intervention Coherence (The Extent To Which An Individual Understands The Intervention And How It Works)

1. If you had to explain the VR experience you had today to family or a friend, how would you explain it?
2. Do you think that a VR program like this one, in which people dance, would help people do more exercise? Why or why not?
3. What is the difference or is there no difference in the VR dance experience you had today vs a typical dance class?

Wearable device questions

1. What did you like most/least about wearing it? Any discomfort or technical difficulties encountered?

2. *[Show them graphic of info Fitbit collects]* Would this motivate you to exercise more? If yes, how exactly does it motivate you? (e.g. step count, calories burned, heart rate zone, etc?

3. *[Create preface]* How would you feel about sharing this information with your study team? Why or why not?

4. Do you think it would be important to include as part of an exercise program?

Opportunity Costs (The Extent To Which Benefits, Profits, Or Values Must Be Given Up To Engage In The Intervention)

1. Imagine you were participating in a VR program to help you exercise more. What other commitments or responsibilities would you have to set aside to participate

a. How else could you be using the time required for the intervention?

Perceived Effectiveness (The Extent To Which An Intervention Is Perceived As Likely To Achieve Its Purpose)

1. What specific aspects of the VR program, if any, did you find effective to promote physical activity?

    a. **Follow-up:** How do the effective aspects align with cultural preferences or Practices?

2. Would you do VR again for physical activity? Would you recommend it to someone else? Why or why not?

    a. **Follow-up:** Do your preferences for doing/not doing VR for physical activity align with the Latino community? b. Follow-up: Would a VR program with Latin dance be helpful to promote physical activity?

3. Would a VR program with Latin dance be helpful to promote physical activity?

Self-efficacy (The Participant's Confidence That They Can Perform The Behavior Required To Participate In The Intervention)

1. Now that you have completed a VR dance program, how confident do you feel to be able to participate in a VR dance intervention (with Latin music) to help you exercise more at home? Why?
2. What helped you be more confident What made you less confident?
3. If someone gave you all the equipment needed to complete a similar program at home, how confident would you feel and why?

Final Question

1. Before we wrap up, is there anything else that someone would like to bring up/mention that didn't come up in the discussion?

Facilitator: "We have had a great discussion, and your contributions have been incredibly valuable. Does anyone have any final thoughts they would like to share before we conclude? Lastly, I want to remind you that we greatly value your privacy and confidentiality. Our discussion today will be used to help develop a virtual reality program that is both interesting and beneficial. Thank you for your participation. We truly appreciate it."

<u>Closing</u>

Post Survey Reminder – ask participants to complete

Facilitator: “Thank you all for your valuable contributions today. As a token of our appreciation, you will receive $100 gift card in visa, you can use it as a credit card. If you have any questions about the study or the next steps, please feel free to ask now or contact me after the session. Have a wonderful day!”
